# Walking along the road with anonymous users in similar attributes

**DOI:** 10.1371/journal.pone.0201532

**Published:** 2018-08-03

**Authors:** Xingchao Bian, Lei Zhang, Lili Yu, Binli Zhang

**Affiliations:** 1 College of Information Engineering, Suihua University, Suihua, China; 2 College of Information and Electronic Technology, Jiamusi University, Jiamusi, China; University of Colorado Denver, UNITED STATES

## Abstract

Recently, the ubiquitousness of smartphones and tablet computers have changed the style of people’s daily life. With this tendency, location based service (LBS) has become one of the prosperous types of service along with the wireless and positioning technology development. However, as the LBS server needs precise location information about the user to provide service result, the procedure of LBS may reveal location privacy, especially when a user is utilizing continuous query along the road. In continuous query, attributes of the user are released inadvertently with per-query, and the information can be collected by an adversary as background knowledge to correlate the location trajectory and infer the personal privacy. Although, a user can employ a central server (CS) to provide privacy preservation for his location, the trustfulness of CS still is without testified and it is usually considered as an un-trusted entity. Thus, in this paper, the trustfulness of CS is verified by a game tree, and then with the result we propose a hash based attribute anonymous scheme (short for HBAA) to obfuscate the attributes released in each query along the road. With the help of HBAA, the CS has no opportunity to get any information about the user who sends his query for generalization service. Furthermore, as the set of attributes is transmitted into a fixed length of hash value, the processing time that spent in attribute generalization is stripped down and the performance of executive efficiency is improved. At last, security analysis and simulation experiment are proposed, and then results of security proving as well as simulation experiments further reflect the superiority of our proposed scheme.

## 1 Introduction

Nowadays, the development of wireless and positioning technology has made devices such as smartphones and tablet computers able to provide location based service, and LBS has become one of the most important parts in people’s daily life. In this type of service, the user can obtain a series of convenient results such as navigation, point of interest providing as well as coupons pushing. However, as the user has to provide precise location information with his service requirement to the LBS server, it is unavoidable to confront the threat of personal privacy leakage. More seriously, the leakage of personal privacy may not only bring about the inconvenience in daily life, but also result in some other serious personal injuries such as tracking, robbery and so on. According to some statistics, there are more than 34% users decide to avoid utilizing LBS and more than 18% users choose to shut down the service in order to prevent privacy leakage[1]. Therefore, privacy leakage has become one of the most serious obstacles to the development of LBS.

In order to cope with the problem of privacy leakage, algorithms such as *k*-anonymity [2], *l*- diversity [3], *p*-sensitivity [4], private information retrieval [5] as well as users correlation [6]were proposed in the past few years. At present, led by schemes of user defined private grid [7], the random walking [8], the personalized query exchanging [9] as well as dummy location distribution [10] and so on has further improved the privacy preservation ability. However, with the development of LBS, more people began to use continuous query instead of snapshot query. This kind of situation leads the above mentioned algorithms difficult to provide privacy preservation for the user, as they were mainly designed for snapshot query. Generally, the continuous query is a type of LBS that provides query result along the movement of the user. In this type of service, the user just needs to send the query once and receives continuous result along his routing with a given time interval or distance. Undoubtedly, this service is more convenient than snapshot query, as it just sends the query once. However, as the continuous query only needs the adjustment of location information, the attribute of the user does not need to be changed, which makes each location can be correlated together and constitutes location trajectory, and this trajectory can be inferred or mined to discover more information about the user. So it will be even easier to get personal privacy of the user when using this type of service.

As the privacy problem of continuous query becomes apparent, the relationship of correlating each location in per-query has been gradually regarded [11–13]. In general, discrete locations are difficult to be correlated into a location trajectory, especially that the locations are generalized. Accordingly, the adversary has to discover the difference of each location and utilize the similarity of attributes to establish the correlation. In order to cut off the correlation of attributes, Palanisamy et al. [14] proposed a delay tolerant mix-zone to generalize the time interval and the velocity movement, and they utilized a non-rectangular mix-zone to achieve above attributes obfuscation. Hwang et al. [15] considered the conception of the synchronization anonymity of time interval, segment, location and trajectory, and provided obfuscated algorithm to generalize these attributes to weaken the correlation. Zeberga et al. [16] hid the attributes with the help of a secure region, and in this region each attribute is disposed with attribute obfuscation, so as to reduce the probability of location correlating in continuous query. Zhang et al. [17] proposed a profile attribute generalization model to choose anonymous users with the maximum comparability, so the similarity of each anonymous user alleviates the correlation between each query. Then Zhang et al. [18, 19] extended the definition of attributes by the consideration that the correlation probability could be another type of attribute, and proposed a correlation probability indistinguishable scheme to prevent the attribute correlation. As the calculation of attribute generalization is costly for the CS, Zhang et al. [20] utilized the CP-ABE to select the collaborative user to achieve attribute generalization. In this algorithm, the CS is just used to expand the anonymous range, and the anonymous user with similar attribute is selected by the ability of decryption. Similar to above algorithm, Zhang et al. [21] completed the private shortest path calculation without any attribute to be observed in cloud environment with the help of homomorphic encryption.

However, although above algorithms can provide privacy preservation service to the user, there still leaves several problems unsolved. For one thing, the attribute generalization is processed by the CS, which does not be verified as a trusted entity, whether it can attack the user to get personal privacy or not cannot be verified. In addition, the CS is just assumed as an un-trusted entity, and there is no rigorous mathematical proof or quantification analysis. For another, most of the operation of anonymous users finding and the comparison of the similar attribute is processed by the CS. Although several methods are used in alleviating the calculation cost, the amount of data processing still exceeds the ability of a common server. Thus, a tradeoff between the user and CS had to be considered, so that the CS can provide privacy preservation service smoothly and efficiently. Then based on the conception of solving above two problems, in this paper, a game tree was first given to analyze the probability of aggressive behavior as well as service providing aspiration of three entities in privacy preservation. Secondly, a hash based attribute generalization scheme was given to achieve the similar anonymous users finding. This scheme is based on the feature of a hash value which is fixed and with strong collision resistance, so it can resolve the problem of CS attempt to get the personal privacy when the user requires for attribute generalization. At the same time, as the comparison of hash value is easier than comparing for each attribute, this scheme can also alleviate the computational cost of the CS. At last, the results of simulation experiments which restricted under the same parameters and compared with other similar schemes were provided, and they demonstrate that the superiority of HBAA is not only on the capability of private preservation but also on the efficiency of execution.

The rest of this paper is organized as follows. Section 2 presents the preliminaries of this paper. The HBAA scheme together with security analysis is described in Section 3. Finally, Experimental results were shown and conclusions were drawn in Sections 4 and 5 respectively.

## 2 Preliminaries

### 2.1 System architecture

**Fig 1:**
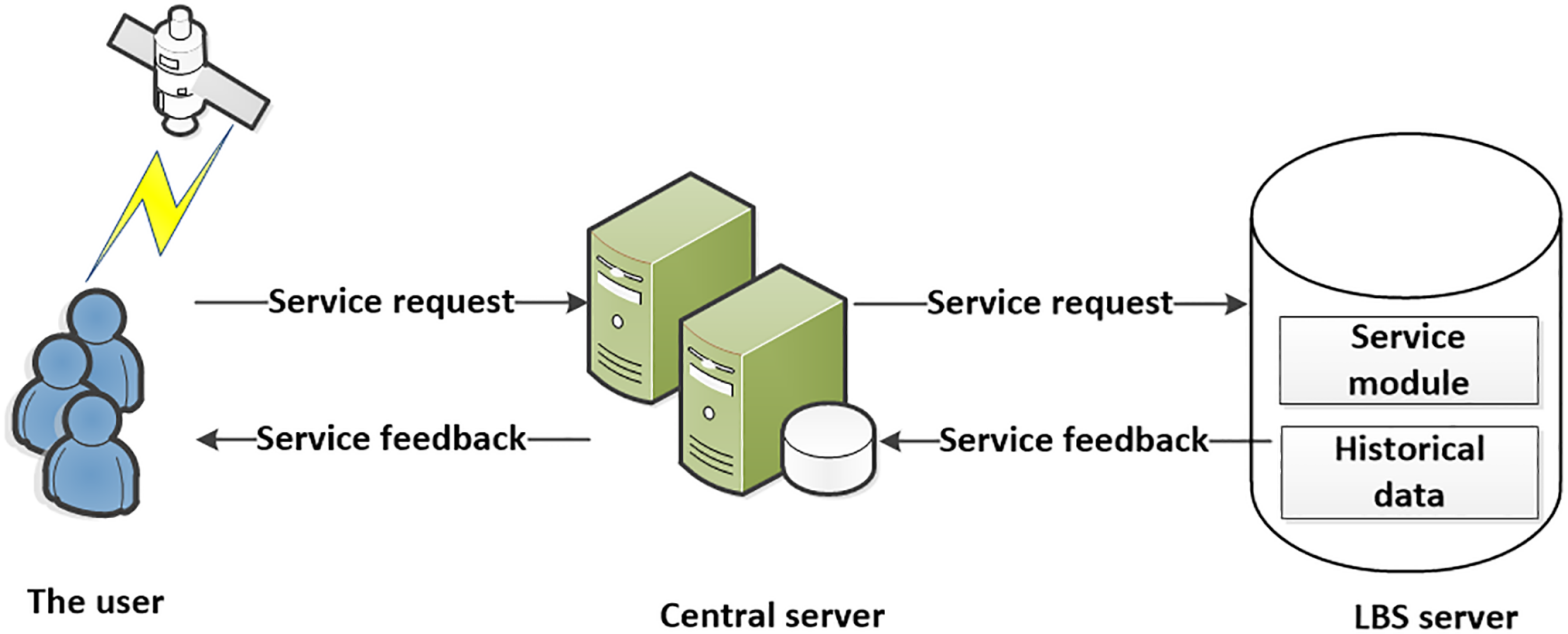
The system architecture of centralized model.

As the operation of attribute generalization is a procedure of calculation cost, the privacy preservation architecture usually employs centralized model. This system architecture is denoted in Fig 1.

In this architecture, three entities are contained, and they are called as user, CS, and LBS server (LBSs) respectively. Among these entities, user refers to the user who equipped with the location and wireless communication device and can send the query information that contains the precise location to the CS or LBSs. The user can also refine the query result from the feedback set of the CS or LBSs to find out his needing. The CS can be seen as an infrastructure that provides privacy preservation service for the user who requires for LBS, so it is usually provided by the research institution, the government department or large enterprises. At last, the LBSs can be seen as the LBS provider, and he can provide navigation, point of interest or coupons pushing service results to the user with the query information and the precise location. Thus, based on this architecture the user can obtain the location based service with personal privacy preservation.

### 2.2 Potential privacy threats analyzed by the game tree

In general, the assumption of centralized system architecture can be depicted as follows. The CS is a trusted entity, as it is provided by some credible enterprises or government, and he can process the information sent from the user without suspicion. The LBSs can be seen as a semi-trusted entity, and he can provide the query result to the user from the information that stored in his server or stored in cloud. But he will be curious about the privacy of the user and may infer the personal privacy with the received query and the background knowledge. However, above assumptions are just imagined by some researchers and there still no proves or quantitative analysis to prove these assumptions. Thus, in this paper, based on the conception of the game tree, a quantitative analysis is given to infer the probability of attacking or service provision. Finally, with the result of the quantitative analysis, the un-trusted entity, attack model as well as the attack technique can be determined, and then the privacy preservation strategy will be formulated accordingly.

**Fig 2:**
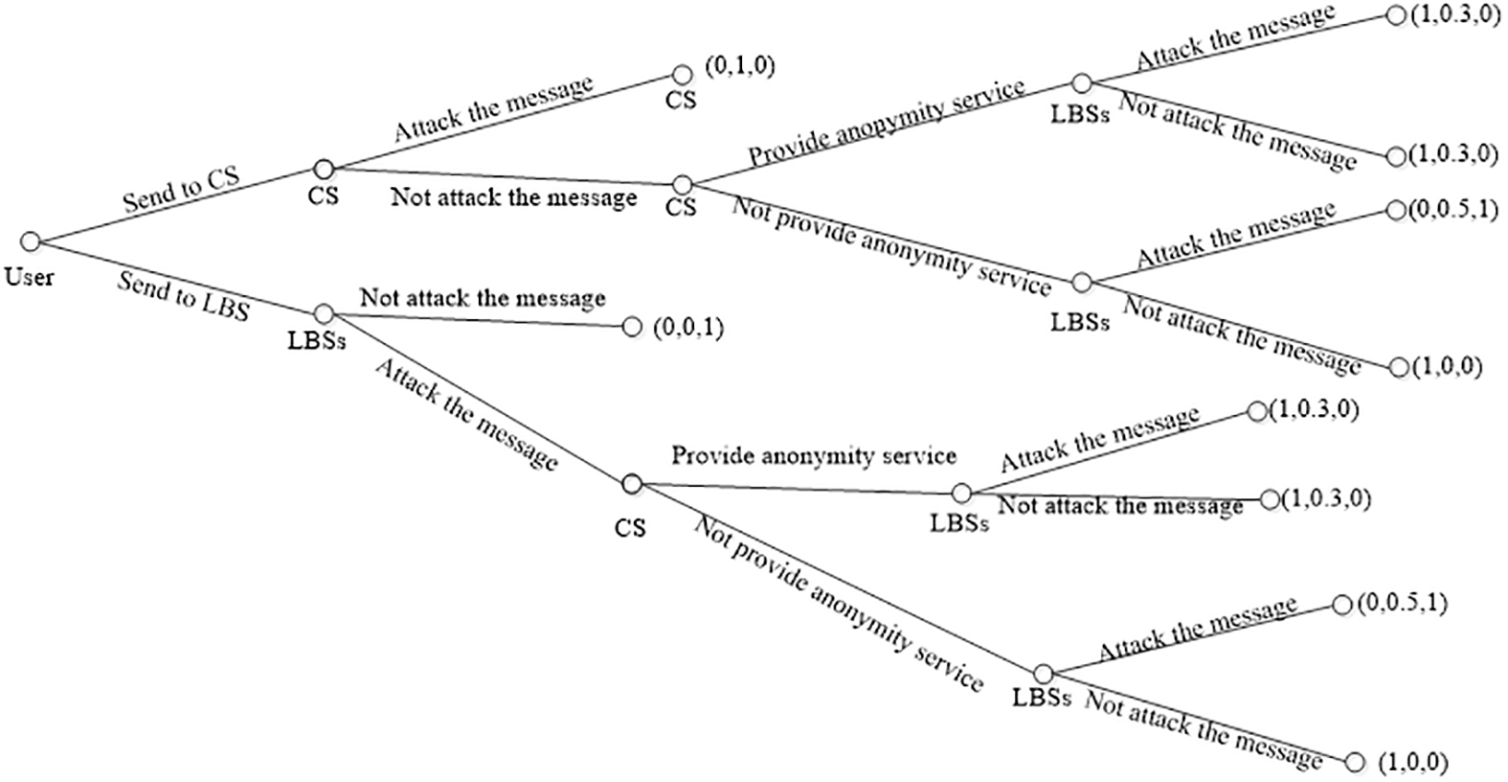
The earnings of three entities in the trilateral game.

Consider that, three entities the user, CS and LBSs are contained in a trilateral game. In this game, the earning of the user is 1 if his privacy is preserved, otherwise, the earning is 0. Earning of the LBSs is 1 if he gets the personal privacy of the user, otherwise, the earning is 0. The earning of the CS seems a bit complex. Like the LBSs, if the CS gets personal privacy from the user, his earning is 1 otherwise the earning is 0. If the CS provides privacy preservation service for the User, he can get the earning of 0.5 from the user, but he has to spend 0.2 for the proceeding of service providing. If the CS does not provide service to the user, he gets nothing from the user and the earning equals 0. In the other case, the LBSs colluding with the CS, and the CS does not provide privacy service to the user, then he can get the earning of 0.5 from the LBSs, if the LBSs initiates an attack to the user and gets his privacy, otherwise, the CS gets nothing and the earning equals 0. Based on above assumptions, a game tree is provided in Fig 2 to analyze the potential of attacking.

Based on the game tree demonstrated in Fig 2, the average earning of the LBSs is 0.6 if he initializes the attack for the privacy of the user. Otherwise, the earning will be dropped to 0 if he does not attack. So in spite of the user utilizes the CS to protect the location, the LBSs still has a higher probability to attack the user and get the privacy. For the CS, his average earning is 0.42 if he attempts to attack the user and get the privacy of the user, otherwise the average earning is 0.275. In addition, if the CS colluding with the LBSs and does not provide privacy preservation for the user, he can get the average earning of 0.25. This earning is less than 0.3 for the earning from providing the privacy preservation service to the user. So the CS can be seen as a semi-trusted entity, as he is willing to provide the privacy preservation service for the user, but has a potential to attack the user to gain a larger earning. At last, for the user, it is better to utilize the CS to obtain the privacy preservation service, as the average earning of employing the CS is 0.75, but this value descending to 0.5 if he does not get help from the CS and just sends the query message to the LBSs.

### 2.3 The attack model and privacy preservation conception

As the user has to provide attribute information during the whole continuous query along the road, the attribute information can be collected by the adversary and he will correlate the discrete locations into location trajectory. The set of attributes may be the query content, the interval times, the running speed and some other symbols of the user. So the attack model can be simulated by the distance of attributes emitted from two adjacent locations. Consider that, if two sets of attributes about a user emitted in two discrete locations are *A*{*a*_1_, *a*_2_, …*a*_*n*_} and $A^{\prime }\{a_{1}^{\prime },a_{2}^{\prime },\ldots a_{n}^{\prime }\}$, then attributes distance between two locations can be denoted as $dis(a,a^{\prime })=\frac{\text{min}(a,a^{\prime })}{\text{max}(a,a^{\prime })}$. Where the min(.) and max(.) are the minimum and maximum value of the current attribute. So the similarity degree of two sets of attributes can be denoted as $Sim\left(A,A\prime \right)=1-\frac{\sum_{i=1}^{n}dis(a_{i},a_{i}^{\prime })}{n},\;0\leq Sim\left(A,A\prime \right)\leq 1$. With the value of similarity of attributes shown in each location, the adversary can compare these values and choose the maximum one. At last, the trajectory can be established and used to infer the personal privacy of the user. This type of similar attribute correlation can be defined as the correlation attack.

Based on the result of the trilateral game analysis, we can conclude that the CS has a higher probability to initiate attack for the privacy of the user, and he may also utilize the correlation attack to infer the privacy with the information that the user sending for attributes generalization. Although privacy preservation scheme such as the algorithm of cor-*k* [22], IRDA [17] as well as *ε*-correlation differential privacy [19] can provide attribute anonymity, they are useless in defending attack initiated by the CS. So the privacy preservation scheme has to consider to defend the attack from the CS, rather than sends the information untreated. Furthermore, as calculation burden of comparing each attribute to find the anonymous user is too heavy, the privacy preservation scheme also has to consider the efficiency of similar attributes finding. Thus, based on above analysis and the feature of CS, a hash-based privacy preservation scheme is proposed. With this scheme, the encrypted value can prevent the CS gets the user’s information, and the fixed value can be used to alleviate the cost in the procedure of similar attributes finding.

For the entity called LBSs, as all attributes must be shown to him to obtain the result of LBS, so the method of attributes encryption is useless. Moreover, as the LBSs has stored a large number of historical query information, he has held the biggest amount of background knowledge. So it seems that it is difficult to avoid the correlation attack initiated by LBSs. But in order to obtain the privacy preservation server, the user can get help from the CS, as he is willing to provide the privacy preservation service for the user. From the analysis of correlation attack, we can see that the correlation attack is initiated by the similarity of attribute sets emitted from two adjacent locations, and the adversary such as the LBSs is just utilizing the maximum comparability to guess the real location of the user. Thus, in order to cope with this type of attack, a general method is to generalize the relationship between each location and get rid of the correlation. As the result of the game analysis, the CS is willing to provide the privacy preservation service, so it is feasible that the CS processes the attribute information to achieve attribute generalization. Consider that, two sets of attributes about a user emitted in random two discrete locations are denoted as *A* and *A*', if the similarity of each location can be denoted as *Sim*(*A*, *A*′) = 1, which means for any location the value of similarity equal to each other. As a result, the adversary cannot correlate any location with the help of correlation attack and the location privacy of the user is preserved.

With the analysis of the attack model and the feature of each entity, we can see that the privacy preservation scheme has to satisfy two conditions. The first one is to encrypt the attribute information to prevent the CS gets the privacy of the user. The other is to generalize the attribute set emitted by the user with other anonymous users and achieves the attribute generalization to descend the success probability of the adversary guesses the real location. Therefore, we proposed a hash-based attribute anonymous scheme (short for HBAA) to achieve above aims. Furthermore, as the hash value is difficult to be falsified, this scheme can also resist the impersonation attack. Additionally, if the CS and the LBSs collude with each other, they may know the privacy of the user during the location service procedure, no matter any privacy scheme is used, so in this paper, we assume that there is no collusion between the CS and the LBSs.

## 3 The hash based attribute anonymous scheme

Similar as schemes for attribute anonymity, the principle of HBAA is also utilize the anonymous user who has similar attributes to generalize the real user and utilize the generalized location to affect the probability of adversary guesses the privacy. The utilization of hash is to accelerate the process of finding enough anonymous users and reduce the threat of CS in the process of generalization, so this scheme only failure when there are not enough users available in the current region. In general, the procedure of this scheme is determined by both the user and the CS. Firstly, the user utilizes the hash to encrypt the sensitive information that the CS cannot decrypt. Then the CS utilizes the value of hash to accelerate the process of finding anonymous users and achieve user generalization with these users. Thus, the process of users can be depicted as information encryption, and the procedure of CS architecture can be depicted as the query information sending, anonymous users selecting and the query result feeding back. With this condition, the procedure HBAA can be depicted as follows.

1. The user encrypts his query information with the public key of the LBSs, and transforms his attributes into a fixed hash value, then sends this message to the CS.
2. After receiving the message from the user, the CS compares the hash value with other anonymous users and chooses the user who has the similar attribute to establish the anonymous group, then sends the group to the LBSs.
3. The LBSs searches query results for the received group from his database or cloud and encrypts each result with the public key of each user, and then he feeds the set of results back to the CS.
4. At last, the CS refines and sends the result to each user.

During the whole procedure, all information passed through the CS is encrypted except the anonymous value *k*. So the CS gains nothing about the personal privacy of the user. Furthermore, the message that the LBSs received is generalized by other users who have similar attributes, it will be difficult for the LBSs to successfully guess the real location of the user during his routing, even though the LBSs initiates the correlation attack. The following of this section will focus on the procedure of query message encryption and the procedure of similar attributes comparing. At last, security analysis will be given to demonstrate the capability of private preservation.

### 3.1 The procedure of message encryption

In order to ease the comprehension of HBAA, several notations and assumptions that will be used in the remainder of the paper are provided in Table 1.

**Table 1:** The notation table.

Notation	Description
*pk*_*L*_	The public key of the LBSs
*k*_*u*_	The public key of the user
*k*	The anonymous value
*q*_*c*_	The query content
*t*	The interval time
*l*(*x*_*t*_, *y*_*t*_)	The location of the user in a certain second
*$E_{pk_{L}}$*	The Ciphertext of LBSs
*H*	The hash value
*$E_{k_{u}}$*	The Ciphertext of the user
*r*	The feeds back result

According to the procedure of attributes anonymity, the query message that the user sends to the CS can be denoted as $Q=\{E_{pk_{L}}(l(x_{t},y_{t}),q_{c},t,k_{\text{u}}),H(A),k\}$. If the CS received this message, he selects anonymous users with hash values comparison and chooses at least *k*-1 users with the similar *H*(*A*) value. Then the CS sends the set of anonymous users as well as the set of queries $Q=\{E_{pk_{L}}(l_{1}(x_{t_{1}},y_{t_{1}}),q_{c_{1}},t_{1},k_{{\text{u}}_{1}}),E_{pk_{L}}(l_{2}(x_{t_{2}},y_{t_{2}}),q_{c_{2}},t_{2},k_{{\text{u}}_{2}}),\ldots ,E_{pk_{L}}(l_{k}(x_{t_{k}},y_{t_{k}}),q_{c_{k}},t_{k},k_{{\text{u}}_{k}})\}$ to the LBSs. When received these sets from CS, the LBSs decrypts the message with his private key and refines out each query content and location coordinate, then finds out the result for each query. At last, the LBSs feeds back the set of results $R=\{E_{k_{u_{1}}}(r_{1}),E_{k_{u_{2}}}(r_{2}),\ldots ,E_{k_{u_{k}}}(r_{k})\}$ to the CS. The CS disassembles the set of results and sends $E_{k_{u}}(r)$ back to each user. At last, the user decrypts the received and obtains the LBS result. During the whole procedure of LBS, there is just encryption message that the CS can receive, and the query message that the LBSs received is an attribute generalization message. So the personal privacy information does not reveal to any entity in this architecture, and then with the attribute generalization, the adversary cannot get any privacy with correlation attack.

### 3.2 The procedure of attribute generalization

In this paper, two different entities that may attack the personal privacy are considered. For attacks that initiated by the CS, the message that passes through this entity is encrypted by the public key of the LBSs or users, so it is difficult for the CS getting any information about the personal privacy. For attacks that initiated by the LBSs, the set of anonymous users has generalized each user with the similar attributes, and the similarity makes the adversary difficult to successfully guess the real location, and then difficult to get the location trajectory.

The output set of users is the set of anonymous users that satisfy the demand of the user. With the set of similar attributes, there are at least *k* users sent to the LBSs. It will be difficult for the LBSs to successfully guess the real location of the user, and then it is difficult for the LBSs to get the location trajectory and the personal privacy is preserved.

### 3.3 Security analysis

As the HBAA algorithm is designed for resisting the attack of two entities, its security is depending on the adversary such as the CS and the LBSs are difficult to obtain the personal privacy of the user, so we analyze the security of HBAA in two conditions.

The first condition is considered as the attack initiated by the CS. In this condition, the CS is attempting to get the privacy of the user, as we had analyzed by the game tree. When the CS received the message sent to him, he will attempt to refine the privacy or reconstruct the hash value. However, as the message sent to the CS is either encrypted or transmitted into hash value, only the anonymous value can be obtained by the CS easily. Furthermore, as one of the features of hash value is collision resistance, it is even harder for the CS to reconstruct the hash value. In addition, the result that feeds back from the LBSs is encrypted by the public key of the user, and the query result of the user also difficult to get without the private key of the user. Thus, during the procedure of similar attributes finding, the CS has no opportunity to access any pragmatic information about the user, lets alone the opportunity to get the personal privacy.

Another condition is considered as attacks initiated by the LBSs. In this condition, the LBSs can get the precise attributes of the user, and he can initiate the correlation attack for the personal privacy of the user. Thus, the security of the user is depending on the LBSs failed to successfully guess the real location. For two arbitrary sets about the locations that the LBSs has received, the attributes of these two locations can be denoted as *A* and *A*'. With the correlation attack, the LBSs has to calculate the similarity between these attributes, and the similarity can be calculated by $Sim\left(A,A\prime \right)=1-\frac{\sum_{i=1}^{n}dis(a_{i},a_{i}^{\prime })}{n}$. If the value of similarity equal to 1, it means these two locations can be correlated as a part of the same trajectory, and then the LBSs can infer the personal privacy of the user with this trajectory, so the privacy of the user is revealed. In order to cope with this type of attack, it is better to make each location has the same probability to be correlated with each other. In HBAA, the attribute of the user is generalized with other anonymous users, so for arbitrarily chosen attributes *A* and *A*′, as well as another different attribute *A*″, the similarity between any two locations can be denoted as *Sim*(*A*, *A*′) ≈ *Sim*(*A*, *A*″) ≈ *Sim*(*A*, *A*″). As the CS chooses at least *k* anonymous users to generalize the attributes, the probability of the LBSs successfully guesses the real location of the user turns into 1/*k*, which is difficult to confirm any location to the user.

## 4 Experimental evaluation

### 4.1 Experiments setup and evaluation criteria

In order to verify the capability of the privacy preservation and execution efficiency of HBAA, a series of comparisons are proposed. These comparisons are simulated by Matlab R2017a and implemented on a laptop with Intel Core i5 1.70 GHz CPU, 4 GB RAM and operated on a Windows 7×64 operating system. We use the central part of the BerlinMOD Data Set to generate the events between users in the local map since it covers more users than the edge areas. The hash function we choose to compare the efficiency contains MD2, MD5, SHA-1, SHA-256, SHA-384, and SHA-512. We also select several similar algorithms such as cor-*k* [22], IRDA [17], SACU [20], DGS [7], MobiMix [23] and MNAME [10] to compare the performance, and the optimal one performs the best. The evaluated criteria will focus on the capability of privacy preservation and the efficiency of algorithm performance. The capability of privacy preservation is evaluated by the time of attributes extraction, the indistinguishable degree of anonymous users, the similarity of two randomly chosen locations as well as the correlation relation between two locations. The efficiency of algorithm performance is evaluated by the running time of hash function process the attribute, the running time of anonymity, the running time of the algorithm, the success ratio of anonymity with the number of users as well as the success ratio of anonymity with the value of *k*.

The time of attributes extraction is calculated by the time of refining attributes from the user’s query sent to the CS. The indistinguishable degree of anonymous users is calculated by the probability of adversary correlate the uncertainty of locations, and this probability can utilize the value of entropy to demonstrate the uncertainty. Assume *A* is a random set of all attributes that the CS sends to the LBSs, as the background knowledge of the adversary has preserved, an attribute set denoted as *A*', then the probability of the adversary successfully guesses the real location can be denoted as *p* = *Sim*(*A*, *A*'). Based on the above probability, the uncertainty of the adversary guesses the real location can be denoted as

$$H(i)=-p(i){\text{log}}_{2}p(i)$$ (1)

Where the larger value of entropy means the more uncertainty. The similarity of two randomly chosen locations is calculated by the probability of two sets of attributes and denoted as *p* = *Sim*(*A*, *A*'), this value changes with the increasing of users. The correlation relation between two locations is calculated by the pair entropy, different from the uncertainty of the adversary, the pair entropy is used to measure the difficulty of the adversary correlates any two randomly chosen locations.

For measuring the efficiency of algorithms performance, the running time of hash function processes the attribute is calculated by a fixed length of the attribute with the increasing number of attributes, so the value changed with the increase of attributes number. The running time of anonymity is calculated by the time of the CS processing the algorithm with the increase of requiring users. The success ratio of anonymity is a bit complicated, as it changed with the increase of requiring users as well as the increase of different anonymous value, so in the comparison the anonymous value is fixed as 30 with the requiring users increasing and the number of requiring users is fixed as 200 with the anonymous value increasing. Then we will give the comparison results as well as a brief analysis of the causing accordingly.

### 4.2 Evaluation results and cause analysis

**Fig 3:**
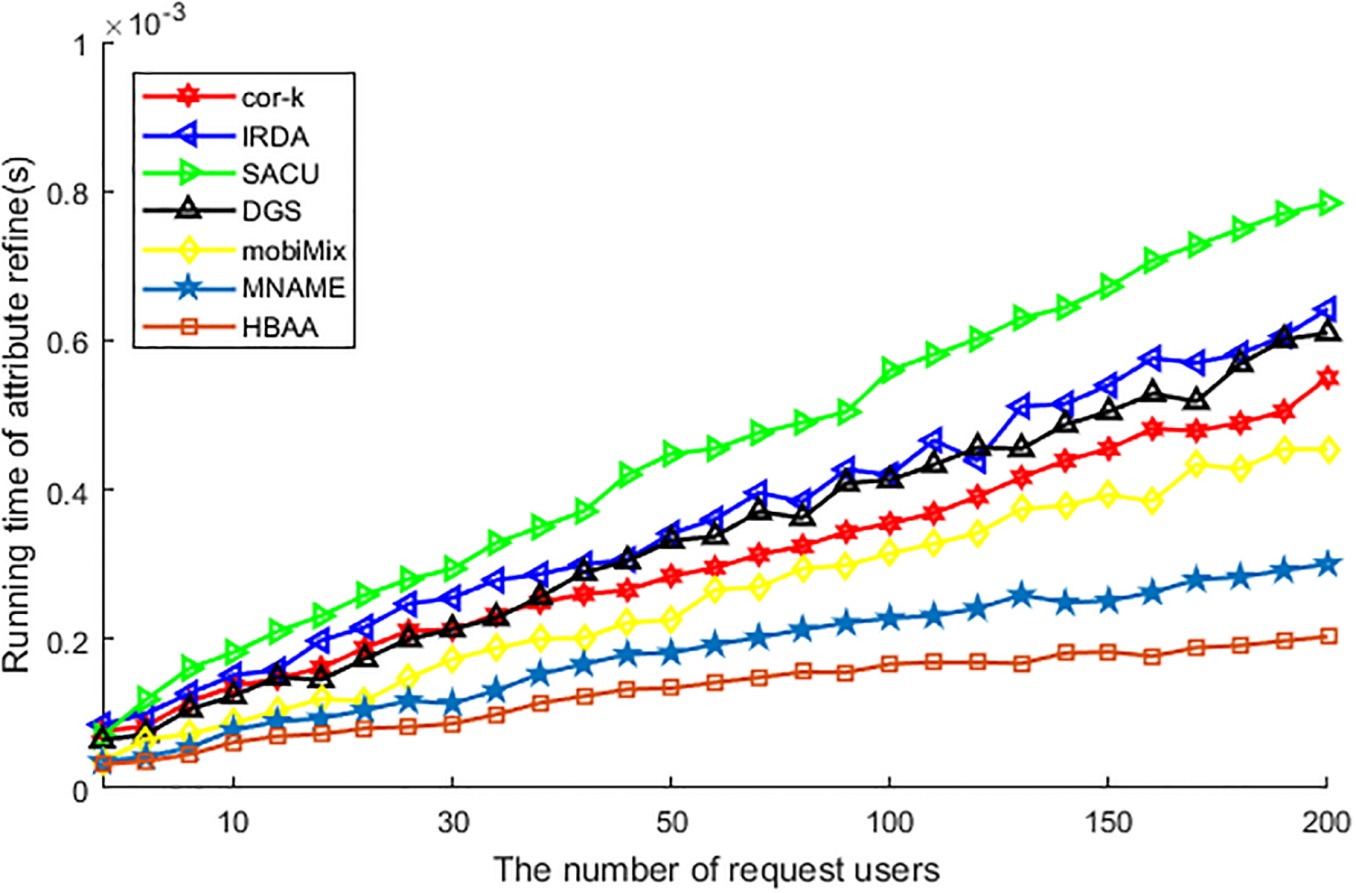
The running time of attribute refine vs. user number.

Fig 3 shows the difference of various algorithms in the time of attributes extraction. From this figure, we can see that the time of SACU is longer than all the other schemes, as this algorithm has to find the collaborative users who want to be concluded in the anonymous group, and the user has the similar attribute to the initiator. This procedure has led to a process of time cost for the attributes refining, and the extraction time was extended. IRDA also has to refine each attribute from the anonymous user, but this algorithm assumes a trusted CS that could provide the attributes refining service for the user, so its performance is better than SACU. As the DGS chooses random cells from the grid, it will affect the time of the attributes refining, if there are no anonymous users located in this grid. But this algorithm also does not need to compare each attribute to refine the attribute, so this algorithm performs better than the algorithm of IRDA. For the algorithm of cor-*k*, as it utilizes the correlation coefficient to take the place of each attribute and utilizes this coefficient to compare the anonymous user’s attribute, the time of refining had been reduced and performance better than above-mentioned algorithms. But still higher than others, as the correlation coefficient must be calculated in advance. Among the remaining algorithms, MobiMix utilizes the mix-zone to disturb the location shift and the delay time. Although just two attributes are considered, it’s delaying time also affects the process of the attributes refining, which leads a higher time for refining than the other two algorithms. MNAME is designed for preserving privacy not only the current location but also for historical query probability. Although the time of refining can be improved by the preserved data, it is still a bit higher than HBAA, as HBAA does not need to refine the attribute but just compare the hash value, this process extraordinary improved the efficiency, so it has the least refining time.

**Fig 4:**
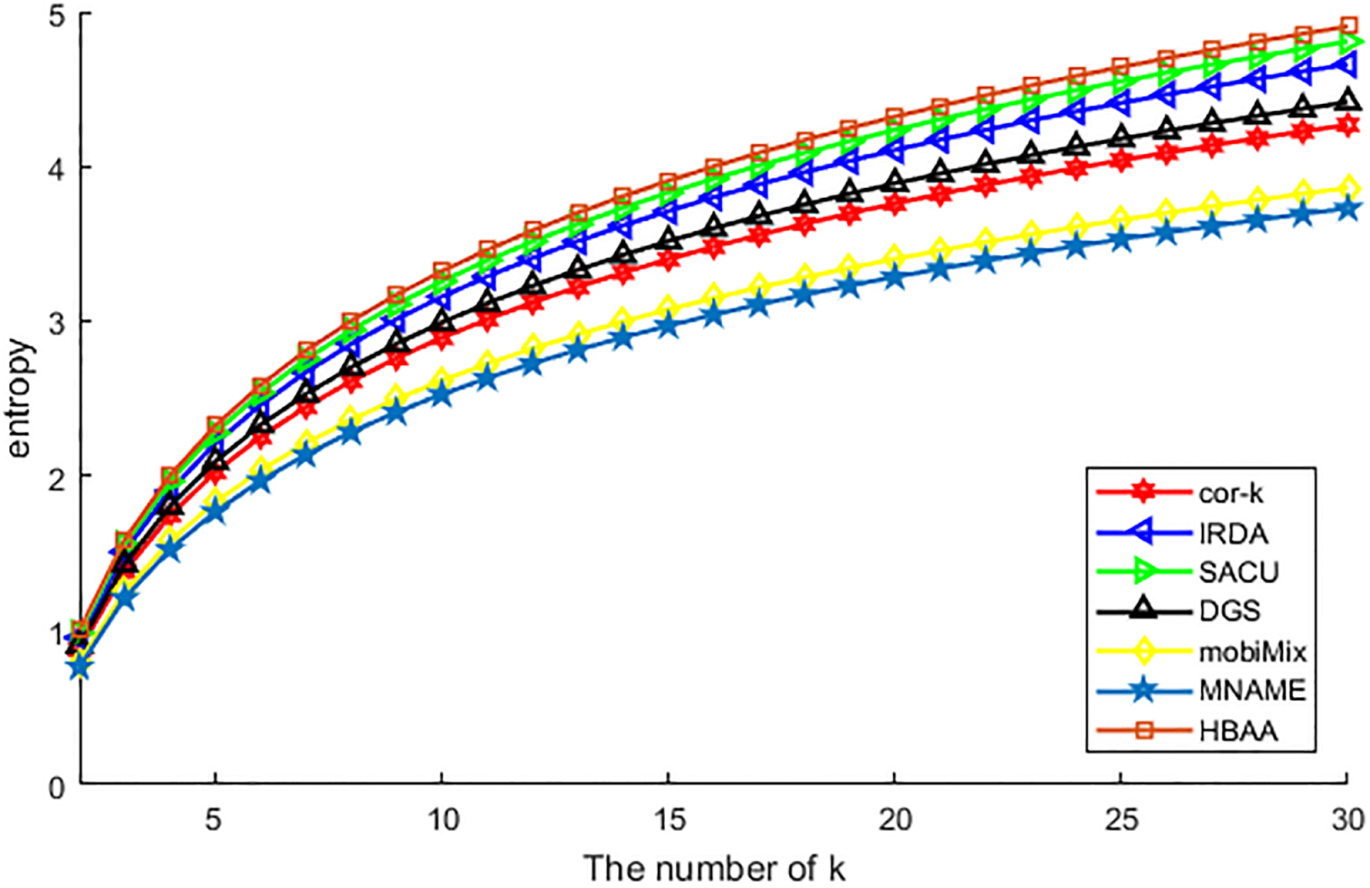
The value of entropy vs. *k*.

Fig 4 shows the difference of various algorithms in the indistinguishable degree of anonymous users. In general, the higher of the value of entropy means the much more uncertainty of the adversary successfully guesses the real location. From Fig 4, we can see that the entropy value of HBAA has reached the maximum value, which means the adversary is different to identify any location in the set of anonymous users. As the HBAA algorithm has utilized the similarity of hash value of the attributes which the user has emitted, each user has the same attributes shown to the adversary, then the adversary is difficult to correlate any location, although utilizing the correlation attack. Among other algorithms, the SACU, the IRDA, and the cor-*k* algorithms are all designed for generalizing the attributes and achieve the purpose of location privacy preservation. The difference of them lies in that, the SACU utilizes the CP-ABE to search for anonymous users with the similar attributes, and the IRDA is choosing these users with a model designed to compare the similarity of each user, at last the cor-*k* is choosing these users with an approximate correlation coefficient. Thus, the values of the entropy of these algorithms can be arranged as that SACU >IRDA >cor-*k*. As designed for hiding the query behind the cell of location grid, DGS has to make certain that the chosen cells contain users with similar attributes to him. But this condition is just a high probability event, sometimes the chosen cell may not contain the user that needed, so is the value of entropy is lower than cor-*k*. At last, algorithms of mobiMix and MNAME just achieve parts of attributes anonymous, and the unconcerned attributes can be used to correlate the real location, so they perform worse than above-mentioned algorithms. But mobiMix is a bit better than MNAME, as it considers more attributes than MNAME.

**Fig 5:**
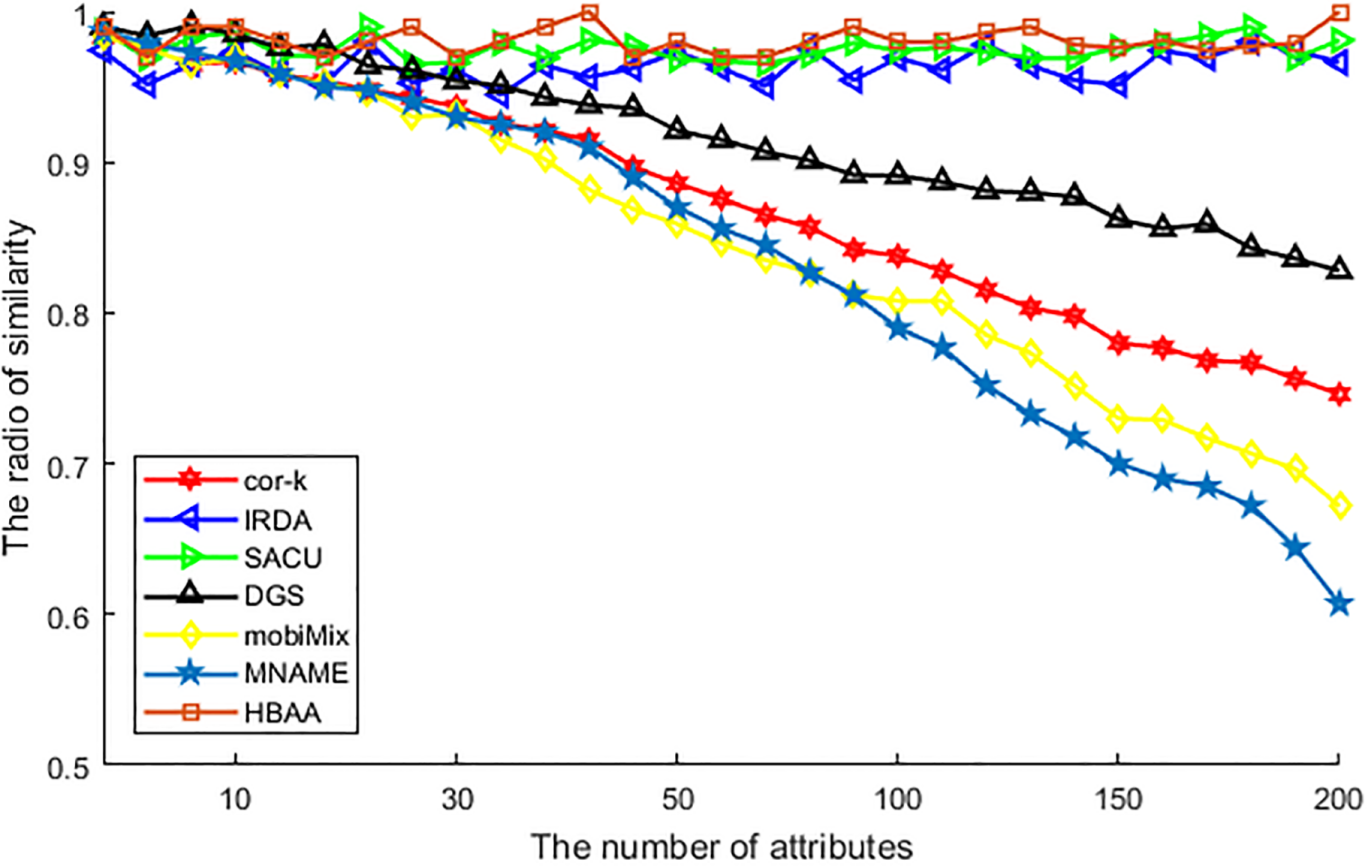
The ratio of similarity vs. the number of attributes.

Fig 5 shows the difference of various algorithms in the similarity of two randomly chosen locations. According to the function of similarity calculation, the ratio of similarity between two locations is calculated by the similarity of attributes. From this figure, we can see that, values of the similarity ratio produced by algorithms of HBAA, SACU and IRDA are similar to each other, as these algorithms are all designed for utilizing attribute anonymity to generalize the correlation of two random locations. Furthermore, as the chosen anonymous users have similar attributes with each other, the similarity of two arbitrarily chosen locations does not be changed with the increase of attributes number. For the other four algorithms, as they are not designed for providing anonymity to all attributes, the similarity is descending with the increase of attributes number. Among these algorithms, DGS performs better than others. This is because of that, this algorithm utilizes grid cells to find the anonymous user with similar attributes, the number of coincident users becomes descending with the increasing of attributes number, and then affects the performance of similarity. The cor-*k* algorithm utilizes correlation coefficient to replace the value of attributes, but this value deviates with the increasing of attributes number, and then the similarity gradually weak accordingly. At last, the mobiMix and MNAME are all difficult in finding enough collaborative user with similar attributes, as they are designed to be used in mix-zone and preserving privacy in the historic location, and the increase of attributes makes the anonymous user even harder to find, so the value of similarity descending. But as the mobiMix consider more attributes than MNAME, the performance of mobiMix is better than MNAME.

**Fig 6:**
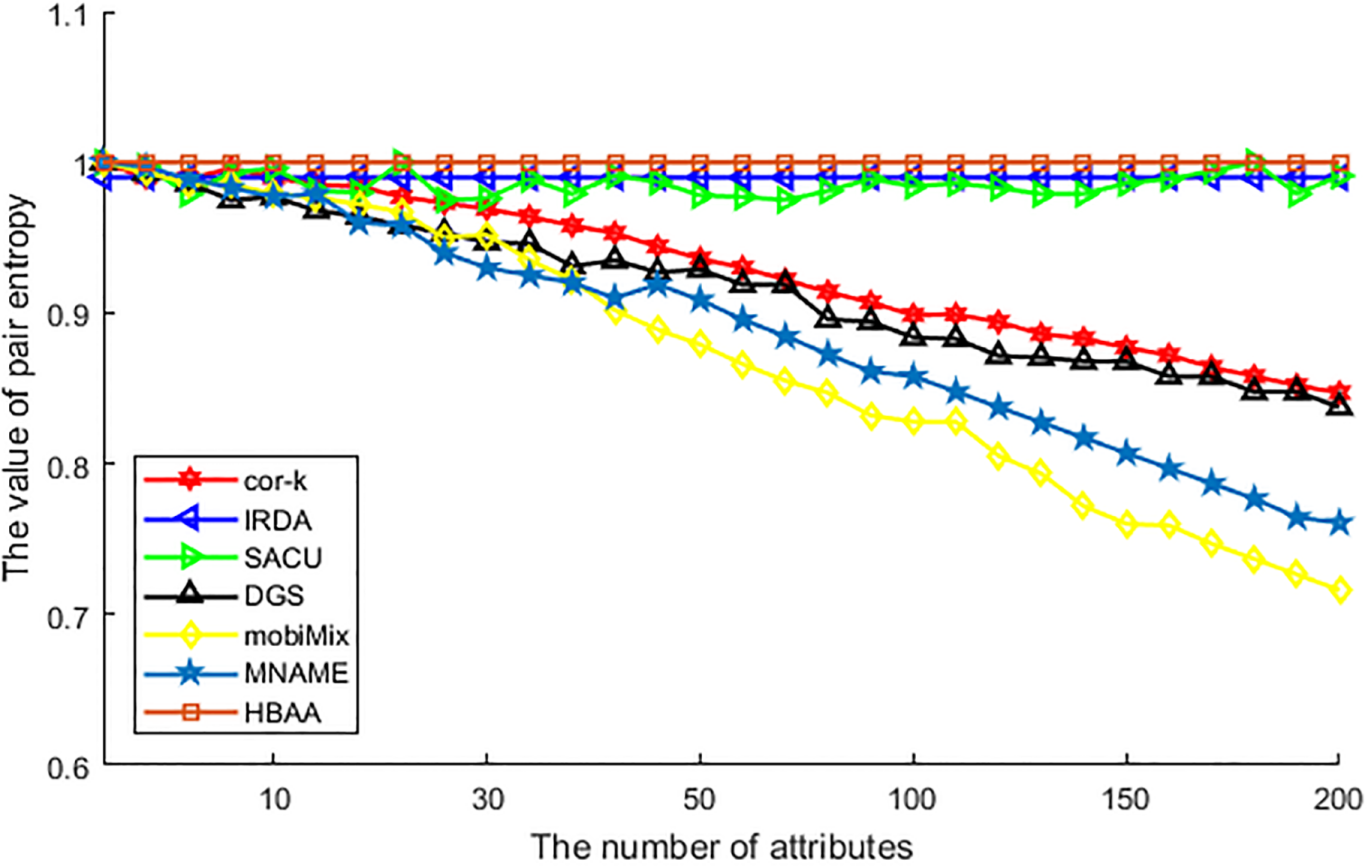
The value of pair entropy vs. the number of attributes.

Fig 6 shows the difference of various algorithms in the correlation relation between two locations. With the values of pair entropy, we can compare the correlation degree of two randomly selected locations, and the larger of this value the less possibility of being correlated with each other. From this figure, we can see that values of pair entropy of HBAA and IRDA nearly all reach the maximum value, as these algorithms can cut off the correlation between two adjacent locations with the attribute similarity. For algorithm of SACU, as this algorithm selects anonymous users with CP-ABE, it is difficult to find enough users to establish an anonymous group, and the variation of choosing enough collaborative users has affected the value of pair entropy, so its value is waving with the increasing of attributes. As other algorithms do not be designed for all potential attributes anonymity, values of pair entropy are all descending with the increasing of attributes. Among these algorithms, cor-*k* utilizes correlation coefficient to replace the value of attributes, but the correlation coefficient becomes deviated with the increase of attributes and leads to a correlative relationship between two arbitrarily selected locations, so the value of pair entropy descending. For DGS, as the consideration of attribute similarity is limited, the relationship between two arbitrarily selected locations is much higher, which makes its value descending. At last, as just a certain number of attributes are generalized, values of pair entropy of MNAME and mobiMix are all descending with the increase of attributes. But as the historic data are easier to be found than real time data, the value of pair entropy of MNAME is higher than mobiMix.

**Fig 7:**
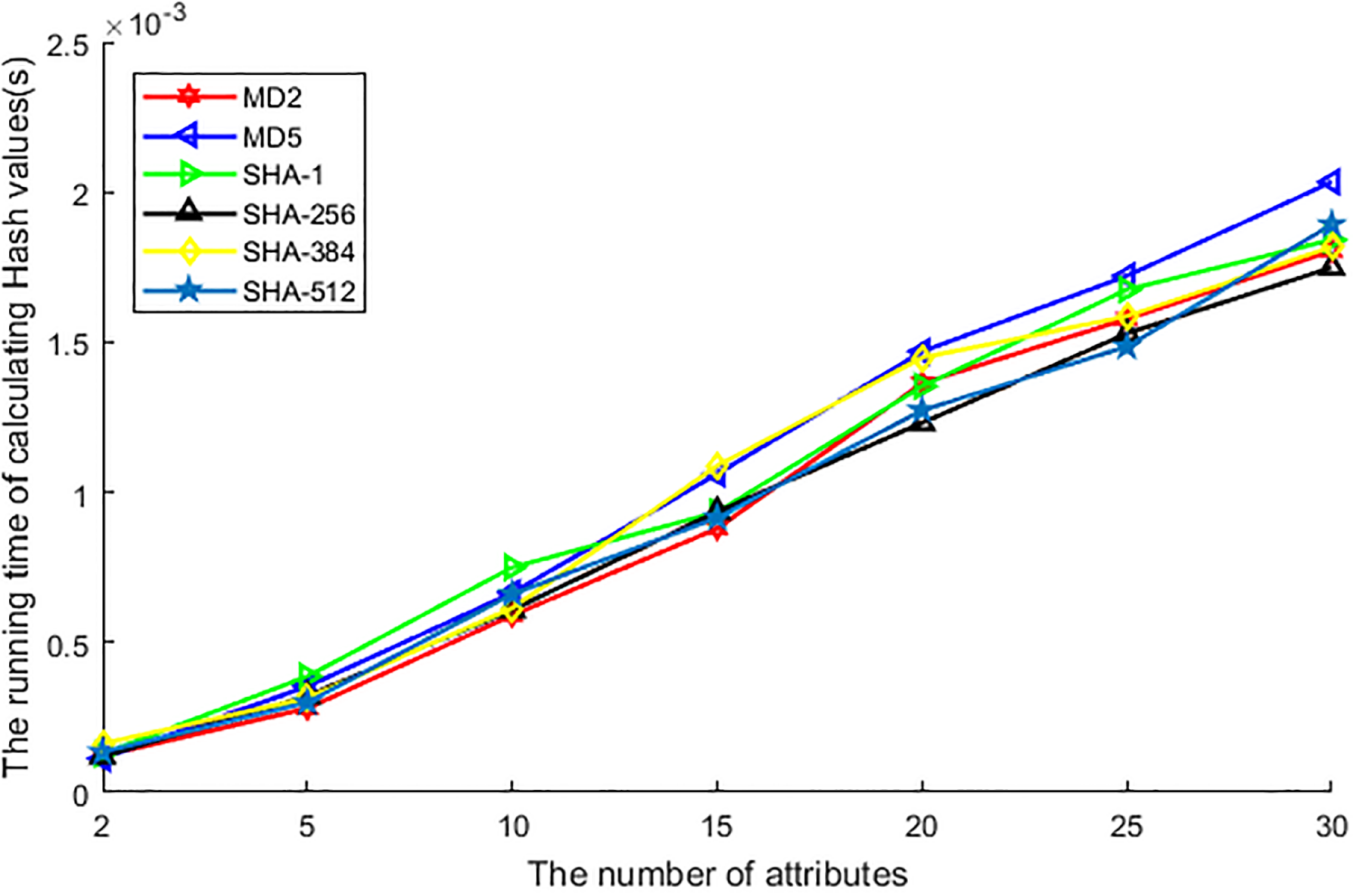
The running time of calculating hash value vs. the number of attributes.

Fig 7 shows the difference of various hash function performance in the running time of processing attributes. Generally, the running time of hash function processes the attribute will affect the performance of HBAA, and a better hash function means a higher level of privacy preservation and higher algorithm efficiency. So which hash function has a lower running time in processing attributes means better to be used in HBAA. In Fig 7, various hash functions with different length of hash value are demonstrated. Although the length of the hash value is different from each other, the running time of processing the attributes seems has just a bit different. However, as the longer the hash value is the more difficult it is to find a suitable value to collide with, it is better to choose the hash function which has a longer hash value. However, as the user has the personalized definition of personal privacy, other hash function with shorter hash value can also be chosen as a beneficial complement for HBAA.

**Fig 8:**
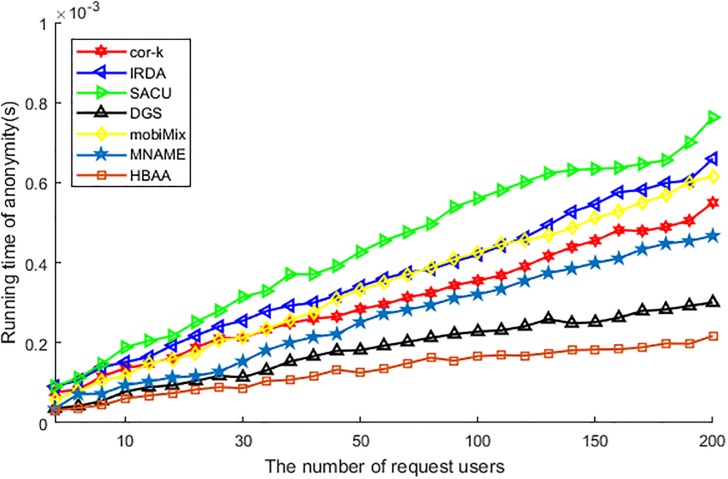
The running time of anonymity vs. the number of request users.

Fig 8 shows the difference of various algorithms in the running time of anonymity. Among these algorithms, the running time of SACU is the longest, as this algorithm has to search for the collaborative user who can decrypt the encrypted query message and wants to be a part of the anonymous group, so its running time of anonymity is the longest. As IRDA needs to compare each attribute with anonymous users to achieve attribute generalization, the process of comparison occupied a lot of time and affects the performance of the running time of anonymity. The mobiMix utilizes delay time to choose anonymous users in mix-zone, this delay also affects the performance of the running time, but the degree of affection is slightly weaker than IRDA. For other algorithms, the cor-*k* has to calculate the correlation coefficient about all attributes, the MNAME has to compare the historic data and the DGS has to choose the cell and prepare the attribute of users, all of these has affected the performance of running time of anonymity, so the running time ascending with the increasing of request users accordingly. At last, as the HBAA just needs to compare the hash value to find the anonymous users, its running time is affected by the user slightly, so in the running time of anonymity the HBAA performs the best.

**Fig 9:**
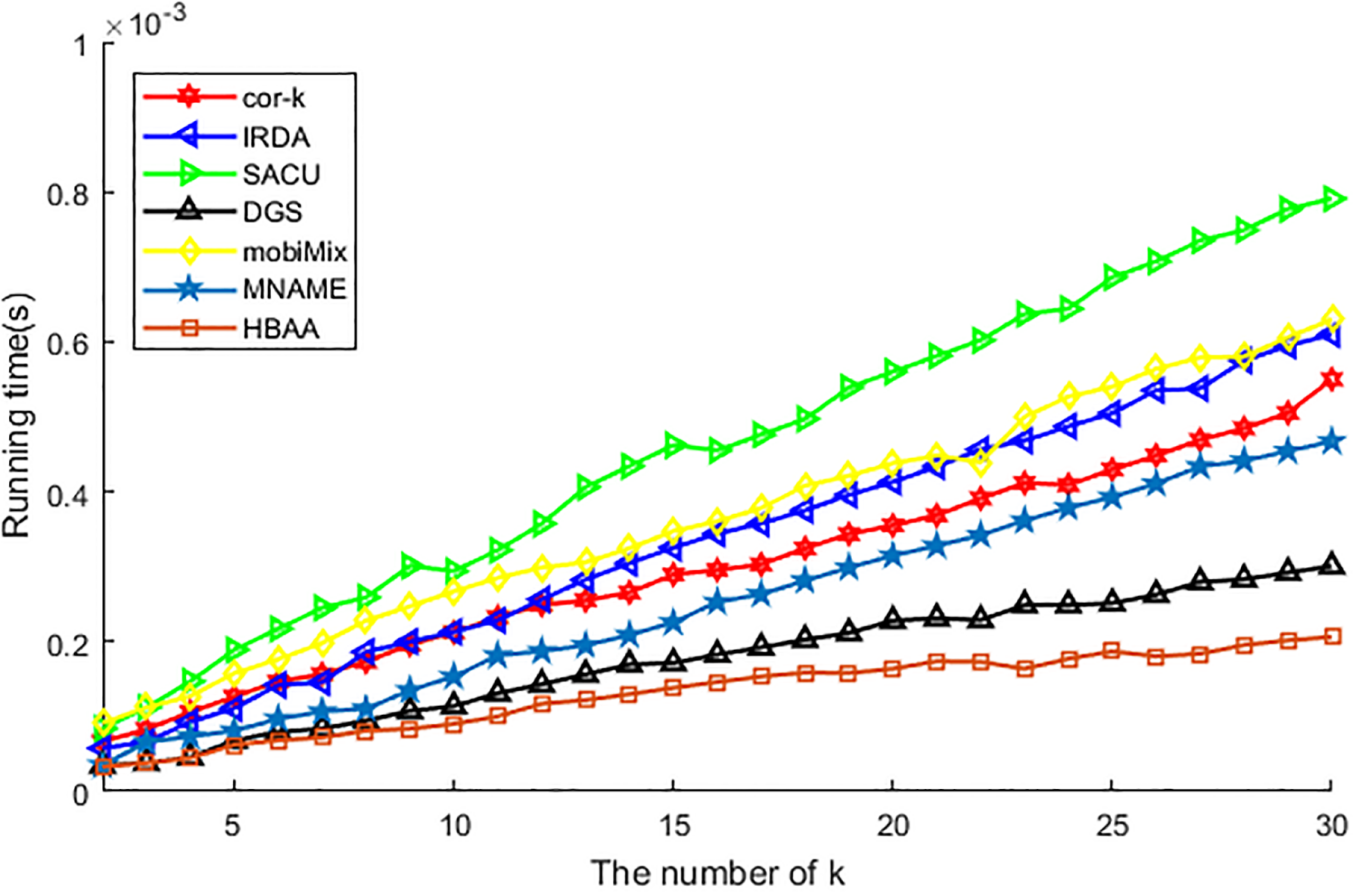
The running time of algorithm vs. *k*.

Fig 9 shows the difference of various algorithms in the running time of the algorithm. Similar to the running time of anonymity, the running time of the algorithm also affected by the difference of processing function. From Fig 9, we can see that, the running time of different algorithms are all ascending with the increase of anonymous value *k*, as the reason this phenomenon is similar to the running time of anonymity. The difference is that the mobiMix is affected by the anonymous value harder than IRDA, so its running time of the algorithm is higher than the latter one.

**Fig 10:**
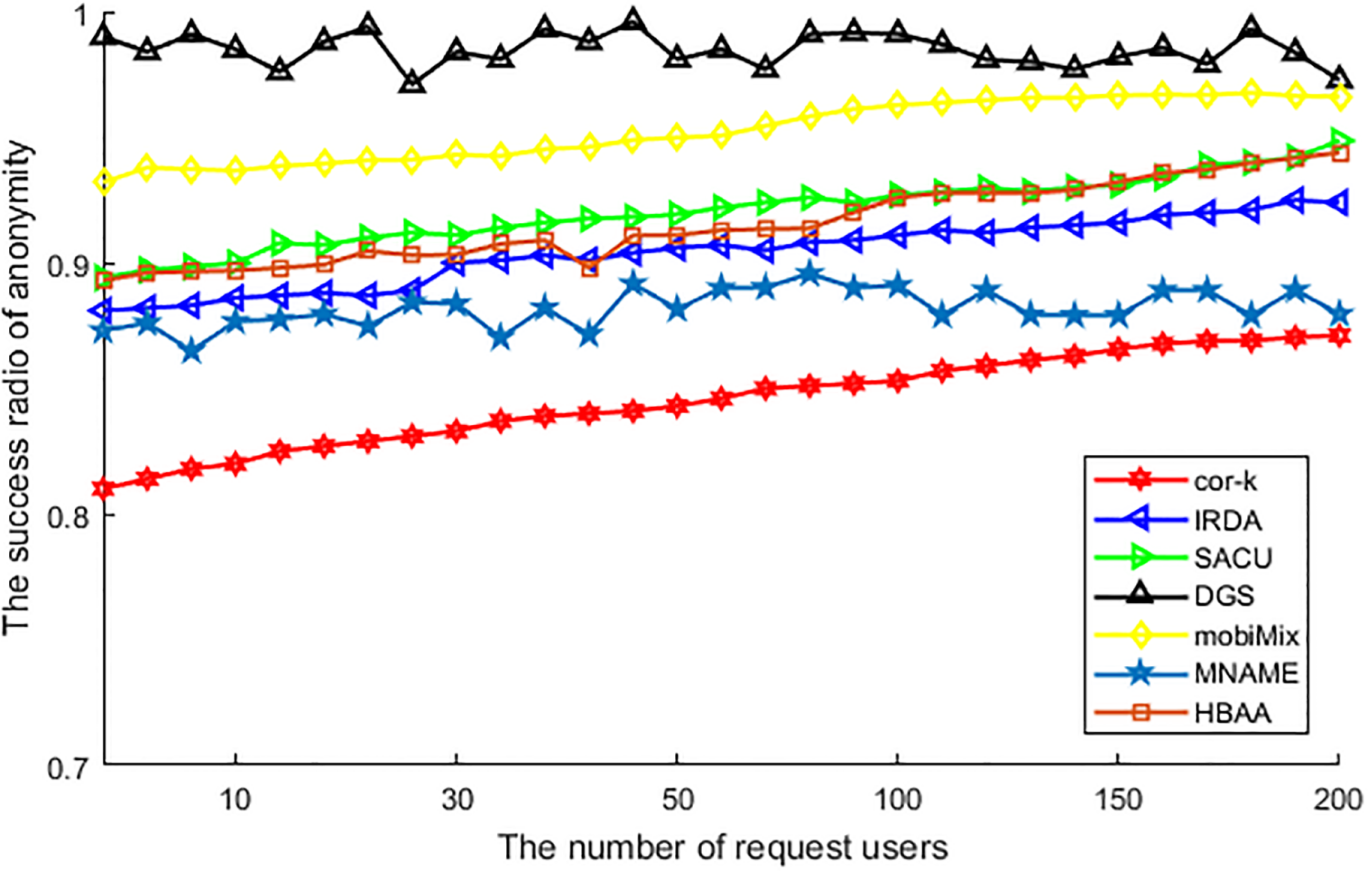
The success ratio of anonymity vs. the number of request users.

Fig 10 shows the difference of various algorithms in the success ratio of anonymity with the number of users. From this figure, we can see that, the success ratio of DGS scarcely affected by the number of requesting users. As this algorithm just utilizes the cell to obfuscate the real location of the user, it is easier to find and choose anonymous users to achieve anonymous group. Thus, the success ratio is higher than other algorithms. For other algorithms, as the increasing of requesting users has provided more users to be involved in the procedure of anonymous users chosen, the success ratio of them is all ascending with the increasing of requesting users. Among these algorithms, the success ratio of mobiMix is higher than the other remaining algorithms. This is because of the mix-zone is usually located in the road segment of a densely populated region, and plenty of populations mean much easier to choose the anonymous users, so it performs better in the success ratio of anonymity. The SACU and HBAA are affected by the comparison of attributes, as some anonymous users in the different attribute will be removed from the anonymous group to guarantee the generalization of attributes. IRDA is affected by the comparison of attribute harder than SACU and HBAA, as it has to compare every attribute but not just the chosen attribute or hash value, so its success ratio performs worse than them. The MNAME also affected by the consideration of historic query probability, and the difficulty of finding a location which has the similar query probability that leads to the failure of success anonymity. At last, cor-*k* is achieved by the comparison of the similar correlation coefficient, and this coefficient can be affected by all attributes as well as the attribute itself, so the success ratio of anonymity about this algorithm is the lowest.

**Fig 11:**
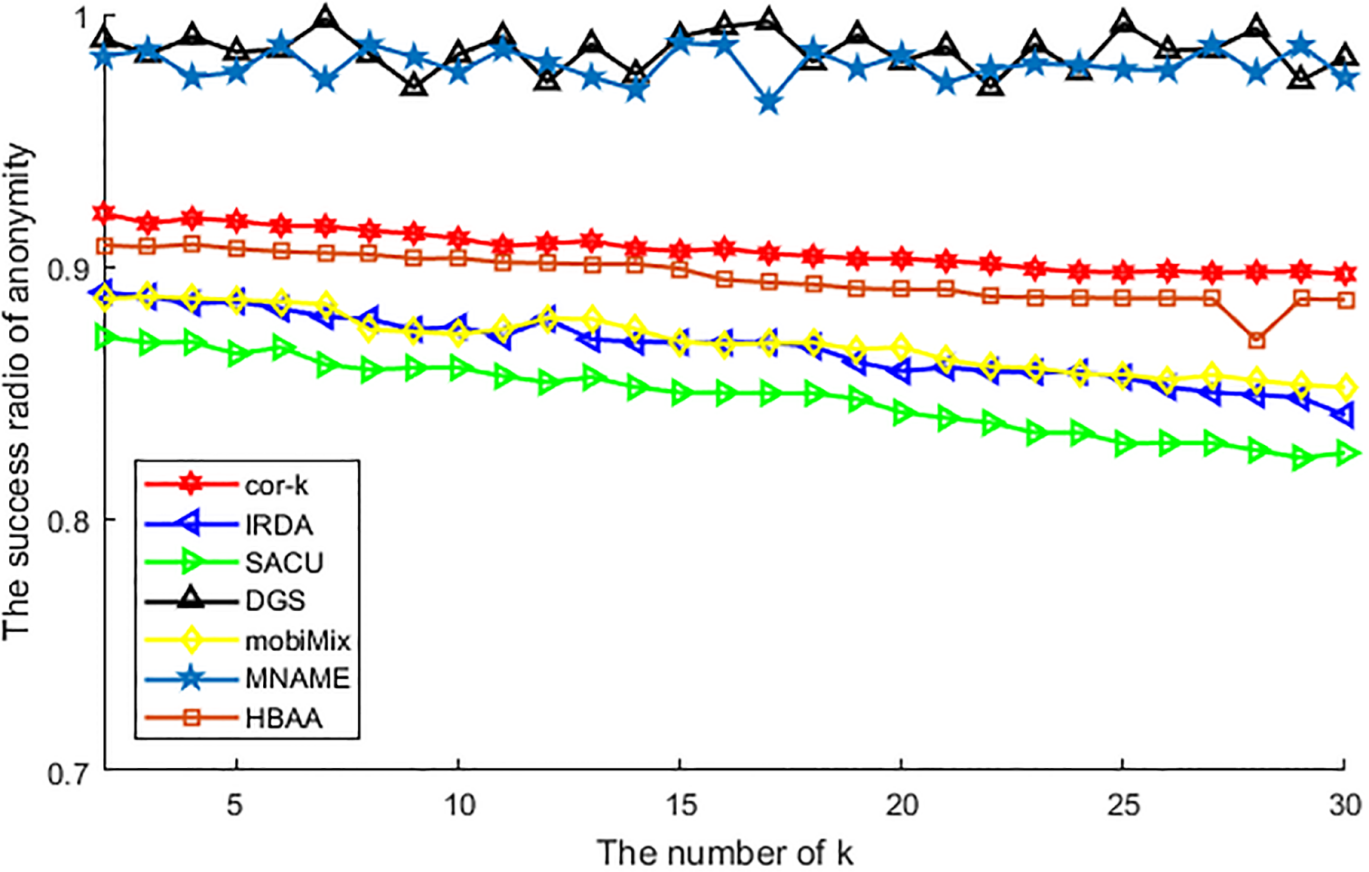
The success ratio of anonymity vs. *k*.

Fig 11 shows the difference of various algorithms in the success ratio of anonymity with the value of *k*. Different from the success ratio of anonymity with the number of users this value does not ascend but descending with the increase of anonymous value. This is because of that, it is difficult to find enough users with the similar attributes to achieve attribute generalization, so the success ratio descending with the anonymous value. From Fig 11, we can see that, algorithms of DGS and MNAME are affected by the anonymous value just a little, as the DGS just considers the number of cells but not the anonymous value and the MNAME is considering the probability of historic query in the current cell, they do not be restricted by the value of anonymous value, so the success ratio of anonymity just waved with whether the cell contains enough anonymous users. For other algorithms, as they all utilized the anonymous users to preserve the personal privacy, so the success ratio of anonymity is descending with the increasing of anonymous value. Among these algorithms, the cor-*k* performs best, as this algorithm utilizes the correlation coefficient to choose the anonymous users, and this procedure makes the CS easier to find enough anonymous users to establish the anonymous group, and then improves the success ratio. The HBAA utilizes hash value instead of the correlation coefficient, so its performance is just a bit failure than cor-*k*, as hash value comparison is more rigorous than correlation coefficient. At last, as the mobiMix needs to wait for anonymous users to establish an anonymous group in the mix-zone, and the IRDA has to compare each attribute with the anonymous user, and the SACU has to confirm the collaborative user who wants to be involved in the procedure of anonymity. So these conditions affect the performance of them, and the success ratio of anonymity is descending with the increasing of anonymous value *k*.

With the results of the comparison and brief analysis, we can conclude that the performance of HBAA is better than other similar algorithms, no matter of capability of privacy preservation or efficiency of algorithm processing.

## 5 Conclusion

When walking along the road with continuous query, the attributes of the user can be collected by the adversary and correlated into location trajectory, then the adversary can infer the personal privacy from this trajectory. In order to cope with this problem, based on the conception of attribute anonymity, this paper proposes a hash-based attribute anonymous scheme. In this paper, the trustfulness of each entity in the system architecture of privacy preservation is firstly analyzed, and with the help of game tree, we proof that the CS has the trend to attack user’s privacy. Then based on the analysis result of the trilateral game, a hash-based attribute anonymous scheme is proposed to resist attacks initiated by the CS and the LBS server. Furthermore, the hash value can also improve the efficiency of similar attributes finding. At last, security analysis and experimental verification were given to further verify the privacy preservation capability of the HBAA as well as its practicability. However, as the technique of attack is continuously developing, some novel attack algorithm will be provided, so the future work will be focused on improving of privacy preservation ability as well as providing privacy preservation service for special environments.

## Supporting information

S1 File The journey data of BerlinMOD. (ZIP)
